# The Relationship between Impulsiveness, Self-Esteem, Irrational Gambling Belief and Problem Gambling Moderating Effects of Gender

**DOI:** 10.3390/ijerph18105180

**Published:** 2021-05-13

**Authors:** Junghyun Choi, Kyoungeun Kim

**Affiliations:** 1 Department of Nursing, Namseoul University, Cheonan 31020, Korea; jhc@nsu.ac.kr; 2 Department of Child Welfare, Namseoul University, Cheonan 31020, Korea

**Keywords:** impulsivity, self-esteem, irrational gambling belief, problem gambling, comparative study, moderating effect

## Abstract

The present study investigated the relationship between impulsivity, self-esteem, irrational gambling belief, and problem gambling and also explored whether the relationships between these constructs are different for males and females. Participants included 563 college students with 259 males (46.0%) and 304 females (54.0%) from Korea. Participants completed a survey. The results showed that 5.3% of students were problem gamblers, while 9.4% were moderate-risk gamblers. The relationships between impulsivity, self-esteem, irrational gambling belief, and problem gambling differed for males and females. For females, greater impulsivity and lower self-esteem predicted higher irrational gambling belief, while higher irrational gambling belief predicted more problem gambling. For males, greater impulsivity predicted higher irrational gambling belief, and higher irrational gambling belief predicted more problem gambling. This finding suggests that different prevention efforts are needed, which will require identifying the variables that affect problem gambling.

## 1. Introduction

Gambling is a common, legal form of entertainment and recreation that is enjoyed by millions of people every day. Most people who gamble can do so without any long-lasting problems or harm. However, gambling can become an addiction. According to recent research, up to one percent of the population is currently suffering from problem gambling [1]. Prevalence studies also indicate that gambling problems could increase in the near future for all populations due to increased access to gambling platforms and increasing gambling opportunities [2]. The size of the gambling industry market in Korea expanded to 20.5 trillion won by 2015 after the government legalized the gambling industry in the early 2000s [3]. In addition, as the gambling industry has become connected to the internet, the accessibility and acceptance of gambling among adolescents have increased. A recent study reported that 28.4% of adolescents in school had gambling experience, while out-of-school adolescents scored 41.8% [4]. The prevalence rates of gambling addiction in Korean adults over 20 years of age were 7.2% in 2012, 5.4% in 2014, and 5.3% in 2018, and they were much higher than England’s 2.5% of 2017 and Australia’s 3.5% of 2017, which was conducted as national scale surveys [5]. Given that gambling can lead to severe adverse consequences and become a progressive disease, many adolescents who have experienced gambling are at risk of problem gambling [6]. In addition, problem gambling results in personal difficulties such as a loss of money, anxiety and depression, suicide risk, and relationship problems [7,8]. For these reasons, studies have been conducted to elucidate the variables affecting problem gambling.

According to Jacobs’ general theory of addictions, there are certain physiological and psychological characteristics or experiences that can make people prone to this form of vulnerability. At a psychological level, pathological gamblers have been found to have lower self-esteem and mood disturbances and participate in risk-taking behaviors to gain excitement and an increase in physiological arousal [9]. Impulsivity, a personality trait which refers to hasty and inappropriate behavior is also considered to influence the severity of gambling-related problem [10,11,12,13]. Impulsive characteristics and gambling-related cognitions are recognized as two major psychological factors in the emergence and retention of problem gambling [14]. A meta-analysis on the relationship between impulsivity and problem gambling showed that intensified impulsive choice, impulsive motor responses, impulsive decision-making, reflection impulsivity, and impulsive cognitive bias are related to gambling disorders; moreover, impulsive decision-making may extend to problem gambling [10]. Impulsivity assessed by the Delay Discounting task, Barratt Impulsiveness Scale, and Impulsive Sensation-Seeking Scale was found to be associated with gambling severity in both clinical and population-based samples [11,13]. Several longitudinal studies have also suggested that childhood impulsivity levels can be a predictive factor for problematic gambling emerging in adulthood [13,15]. Therefore, the model in this study assumed a direct path between impulsivity and problem gambling.

Self-esteem refers to an appraisal of oneself; therefore, low self-esteem is correlated with various types of psychological distress, such as depression and anxiety. A study found that problem gamblers invariably expected others to think of them badly, and these negative social outlooks and personal shame make them feel like failures and lower their self-esteem [16]. On the other hand, many studies have shown that people with low self-esteem are vulnerable to different types of addictive behavior [17,18,19,20,21]. Problem gamblers were found to be more alienated from society, to have lower self-esteem, and to be more likely to participate in various high-risk behaviors than other adolescents. Volberg, Reitzes, and Boles [18] also found a relationship between low self-esteem and problem gambling and between high self-esteem and non-problem gambling. Several Korean studies were conducted on the effects of self-esteem on addiction [19,20,21]. In a meta-analysis study of 29 research papers in Korea, self-esteem was identified as one of the biggest effect size variables related to adolescent gambling behavior [20]. In an explorative study in Korea, self-esteem was one of the significant predictors of a gambling problem among college students [21]. Therefore, the model in this study also assumed a direct path between self-esteem and problem gambling.

Problem gambling is often accompanied by various irrational beliefs that encourage excessive behavior [4]. People who gamble tend to have irrational beliefs, leading to risky decisions and excessive gambling [22]. According to cognitive–behavioral formulations, irrational cognitions play an important role in maintaining problem gambling behaviors [23]. Irrational beliefs in gambling include overestimating one’s probabilities of winning [24], superstitious rituals [25], the gambler’s fallacy [26], and misunderstanding the independence of chance events [27]. For example, a study of 185 college students found that those in the problem gambling group had stronger beliefs on the Good Luck Scale and were more positive about gambling [28]. Among the multiple factors affecting problem gambling, one of the most important seems to be cognitive biases or distortions; these biases, such as dysfunctional decision-making, are thought to lead to problem gambling [29]. Therefore, the model in this study assumed a direct path between irrational belief and problem gambling.

In addition, irrational beliefs appear to play a mediating role in the connection between emotional characteristics (impulsivity and self-esteem) and problem gambling. Several studies show that impulsive adolescents are likely to have more irrational beliefs [30], and failing to resist harmful impulses seems to lead to dysfunctional cognition. In a study of 377 high school students in Korea, irrational beliefs in gambling had a mediating effect on the relationship between emotion-centered coping and gambling addiction. In stressful situations, emotion-focused coping reinforced gambling behavior by increasing irrational beliefs about gambling [31]. In addition, self-esteem was found to be related to irrational beliefs. In a study of 251 university students, low self-esteem was negatively associated with irrational beliefs such as anxious overconcern, high self-expectations, demand for approval, and other problems [32]. Moreover, in a longitudinal study on the causal relationship between gambling beliefs and gambling behavior, gambling beliefs affected gambling behavior five months later, and gambling behavior also affected gambling beliefs five months later. This result indicates that there is mutual causality between gambling behavior and irrational gambling beliefs [33]. These studies support that irrational beliefs can be assumed to mediate the effect of an individual’s emotional characteristics (e.g., impulsivity and self-esteem) on gambling behavior.

While impulsivity, self-esteem and irrational beliefs can be understood as increasing gambling behaviors, the gender differences in the relationships between these variables need to be considered. A systematic literature search published in 2016 showed worldwide gender differences in gambling behavior [34]. The prevalence survey on adult gambling behavior with 929 Cyprians revealed men are more likely to experience gambling-related problems [35]. In the nationwide Finnish Gambling 2011 survey with 4484 Finns, the overall problem gambling prevalence rate was 0.6%, and problem gambling was more prevalent among males [36]. In another National gambling survey conducted in Iceland with 1887 individuals, males were more likely to be categorized as problem gamblers [37]. In addition, according to the First Brazilian National Alcohol Survey, men were 2.3 times more likely to be exposed to gambling than women and 3.6 times more likely to experience gambling-related problems than women [38]. Using cross-lagged panel models, the study found that men had a significantly stronger tracking correlation with gambling urges over time than women when adjusting for cognition paths [39]. In the Korean studies, of 734 boys and girls attending middle and high school in Korea, male students had a higher level of problematic gambling than female students (34.6% vs. 19.7%) [40]. Based on the 2018 survey on youth gambling problems in Korea, the proportion of men in the problem gambling group and the risk-level group was 73.9% and 54.7%, respectively [41]. These studies show that gender differences exist in gambling behavior; however, there have been few studies that verified the relationship between impulsivity, self-esteem, irrational beliefs and problem gambling in males and females. To fill this gap, the present study aimed to determine whether the relationships between impulsivity, self-esteem, irrational gambling belief, and problem gambling are different for males and females.

**Figure 1:**
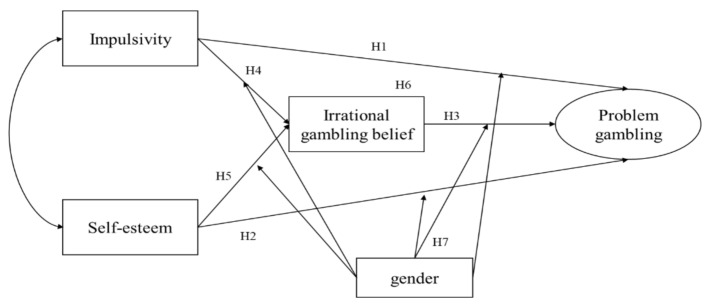
Research model.

The detailed hypotheses of this study are as follows (See the Figure 1):

**Hypothesis** **1** **(H1).** *College students with a higher level of impulsivity are more likely to show problem gambling*.

**Hypothesis** **2** **(H2).** *College students with a lower level of self-esteem are more likely to show problem gambling*.

**Hypothesis** **3** **(H3).** *College students with a stronger level of irrational gambling belief are more likely to show problem gambling*.

**Hypothesis** **4** **(H4).** *College students with a higher level of impulsivity are more likely to show irrational gambling beliefs*.

**Hypothesis** **5** **(H5).** *College students with a lower level of self-esteem are more likely to show irrational gambling beliefs*.

**Hypothesis** **6** **(H6).** *The effect of impulsivity and self-esteem on problem gambling is mediated by irrational gambling beliefs*.

**Hypothesis** **7** **(H7).** *Gender moderates the links between impulsivity, self-esteem, irrational gambling belief, and problem gambling*.

## 2. Materials and Methods

### 2.1. Design

This is a comparative study designed to examine the relationships between impulsivity, self-esteem, irrational gambling belief, and problem gambling among college students in Korea.

### 2.2. Sampling and Data Collection

Participants included 563 college students, with 259 males (46.0%) and 304 females (54.0%) from Korea. The range of age was 17 to 46, and the average age was 20.87 (*SD* = 2.70). The purpose of this study, data confidentiality, and the possibility of withdrawal were explained to the participants, and the written consent of the participants and their parents was obtained in advance.

### 2.3. Measurement

#### 2.3.1. Problem Gambling

Problem Gambling was measured by the Problem Gambling Severity Index (PGSI), which is composed of 9 items from 31 items of the Canadian Problem Gambling Index (CPGI) [42] (e.g., “*How often have you bet more than you could really afford to lose?*, *How often have you needed to gamble with larger amounts of money to get the same feeling of excitement*?). These 9 items were evaluated with a scale from 0 to 3 points (0 points = never, 1 point = sometimes, 2 points = most of the time, 3 points = almost always). The range of the total score is from 0–27. According to the total score, the degree of gambling addiction is divided into non-problem gambling (0 points), Low level of problems with few or no identified negative consequences (1–2 points), Moderate level of problems leading to some negative consequences (3–7 points), and Problem gambling with negative consequences and a possible loss of control (8 points or higher). A higher score indicates a more severe level of Problem Gambling. The Cronbach’s alpha coefficient was 0.94.

#### 2.3.2. Impulsivity

The impulsivity index used by Kim et al. was employed in this study [43]. This tool is composed of 8 items for asking about impulsivity (e.g., “*I can’t sit in one place for a long time because I’m frustrated” and “I’m going crazy if I don’t do what I want to do right away*). Each item was evaluated with a 4-point scale (*1 = Not at all*, *2 = Almost no*, *3 = Almost yes*, *4 = Exactly yes*). The impulsivity score was determined based on the average points of the 8 items. A higher score means a higher level of impulsivity. The Cronbach’s alpha coefficient was 0.87.

#### 2.3.3. Self-Esteem

The self-esteem scale developed by Rosenberg [44] was used in this study. This scale is composed of 10 items asking about one’s degree of self-esteem (e.g., “I have a lot of good points” and “I am a very competent person”). Each item was evaluated with a 4-point scale (*1 = Strongly disagree*, *2 = Disagree*, *3 = Agree*, *4 = Strongly Agree*). A higher score indicated a better level of self-esteem for the student. The Cronbach’s alpha coefficient was 0.84.

#### 2.3.4. Irrational Gambling Belief

In this study, the irrational gambling belief scale was composed of 10 items that were selected by Kwon [45] on the basis of Steenbergh’s questionnaire for Gambling belief [46] (e.g., “*To win a gambling game*, *I should have a good strategy*” and “*My knowledge and skill in gambling contribute to the likelihood that I will make money*”). Each item was evaluated with a 5-point Likert scale (*1 = Strongly disagree*, *2 = Disagree*, *3 = Usually neither agree or disagree*, *4 = Agree*, *5 = Strongly agree*). The score of irrational gambling belief was indicated by the average points of ten items. A higher score indicated a higher level of irrational gambling belief. The Cronbach’s alpha coefficient was 0.91.

### 2.4. Ethical Consideration

This study received ethical approval from the Institutional Review Board of Namseoul University (IRB No. NSU-202004-005).

### 2.5. Data Management and Analysis

To examine the relationship between impulsivity, self-esteem, irrational gambling belief, and problem gambling and to investigate the mediating effect of irrational gambling belief in the relationship between impulsivity, self-esteem, and problem gambling, structural equation modeling (SEM) was used. To explore the moderating effect of gender on the relationship between impulsivity, self-esteem, irrational gambling belief, and problem gambling, a multi-group analysis of structural equation modeling (SEM) was used. First, the causal relationship of the measurement model was estimated. Second, an unconstrained model that does not impose restrictions on the relationships of latent variables was estimated. Third, we estimated an equality-constrained model that assumed the sizes of the relations between variables to be equal to each other. Then, the moderating effects of gender were analyzed by comparing the model fits of the two models. The chi-square goodness of fit statistic was used to assess the degree of model fit and the incremental fit indices, such as the Comparative Fit Index (CFI), Normed fit index (NFI), and Root Mean Square Error of Approximation (RMSEA).

## 3. Results

### 3.1. Descriptive Statistics and Correlation

Table 1 shows the ranges, means, standard deviations, skewness, and kurtosis of the main variables. The average impulsivity scores of males and females were 1.97 and 2.07, respectively. The average self-esteem scores of males and females were 3.00 and 2.86, respectively. The average irrational gambling belief scores of males and females were 2.20 and 1.85, respectively. Males were more likely to have higher self-esteem and more irrational gambling belief than females (*t* = 3.06, *p* < 0.001; *t* = 4.90, *p* < 0.001). However, there was no significant difference in impulsivity between males and females. The value of the skewness and kurtosis of impulsivity, self-esteem and irrational gambling belief supported the assumption of a normal distribution of the data; the absolute value of skewness was less than 3, and the absolute value of kurtosis was less than 10 [47].

Table 2 shows gender differences in problem gambling severity (*χ*^2^ = 23.92, *p* < 0.001). The results showed that 5.3% of the survey subjects were problem gamblers, while 9.4% were moderate-risk gamblers. In male participants, non-problem gamblers were 59.1%, low-risk gamblers were 19.7%, moderate-risk gamblers were 13.9%, and problem gamblers were 7.3%. In female participants, non-problem gamblers were 77.3%, low-risk gamblers were 13.5%, moderate-risk gamblers were 5.6%, and problem gamblers were 3.6%.

Table 3 shows the interrelations between impulsivity, self-esteem, and irrational gambling belief and problem gambling. Impulsivity and irrational gambling behaviors were positively associated with problem gambling, and impulsivity was positively associated with self-esteem and irrational gambling behavior. However, self-esteem was not significantly associated with irrational gambling beliefs or problem gambling.

### 3.2. Model Fitness

**Figure 2:**
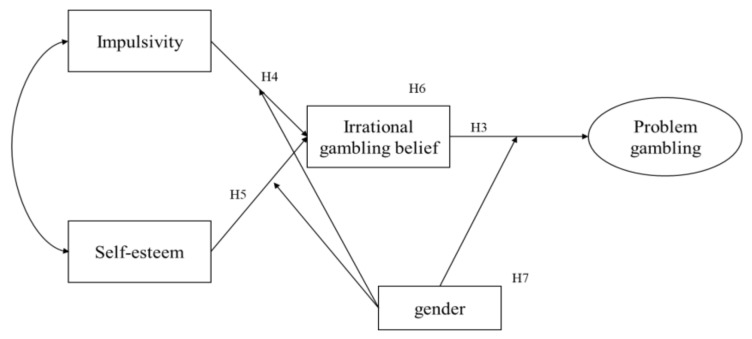
Alternative model.

The hypothesized model of the study is the relationship between impulsivity, self-esteem, and irrational gambling belief and problem gambling for the total sample. Table 4 shows the goodness-of-fit of the hypothesized model. The model fit indexes of the hypothesized model were *χ*^2^ = 46,44, *df* = 6, *p* = 0.00, NFI = 0.97, CFI = 0.97, and RMSEA = 0.80. The path from impulsivity to problem gambling and the path from self-esteem to problem gambling were not significant. To improve the model fit, an alternative model without the path from impulsivity to problem gambling or the path from self-esteem to problem gambling within the hypothesized model was explored (Figure 2). The alternative model showed a better fit than the hypothesized model, as seen in Table 4 model (*χ*^2^ = 51.04, *df* = 8, *p* = 0.00, NFI = 0.97, CFI = 0.99, RMSEA = 0.70). The alternative model was adopted as the final model according to the principle of *parsimony* (Δ χ^2^_*df* = *2*_ = 4.60, *p* > 0.05). Thus, H1 (College students with a higher level of impulsivity are more likely to show problem gambling) and H2 (College students with a lower level of self-esteem are more likely to show problem gambling) were rejected. However, H3 (College students with a higher level of irrational gambling belief are more likely to show problem gambling) and H4 (College students with a higher level of impulsivity are more likely to show irrational gambling beliefs) were accepted.

The regression weights of the final model are presented in Table 5. Impulsivity (β = 0.29, *p* < 0.001) and self-esteem (β = −0.12, *p* < 0.05) had a significant relationship with irrational gambling belief, and irrational gambling belief had a significant positive relationship with problem gambling (β = 0.28, *p* < 0.001).

Estimates of the direct, indirect, and total effects of impulsivity and self-esteem on problem gambling via irrational gambling belief are shown in Table 6. Impulsivity (β = 0.08, *p* < 0.01) and self-esteem (β = 0.03, *p* < 0.05) had an indirect effect on problem gambling via irrational gambling belief. College students with higher impulsivity and lower self-esteem were more likely to have greater irrational gambling beliefs, and college students with greater irrational gambling beliefs were likely to have a more severe problem gambling. H6 (The effect of impulsivity and self-esteem on problem gambling is mediated by irrational gambling belief) was accepted.

### 3.3. Multi Group Analysis

**Figure 3:**
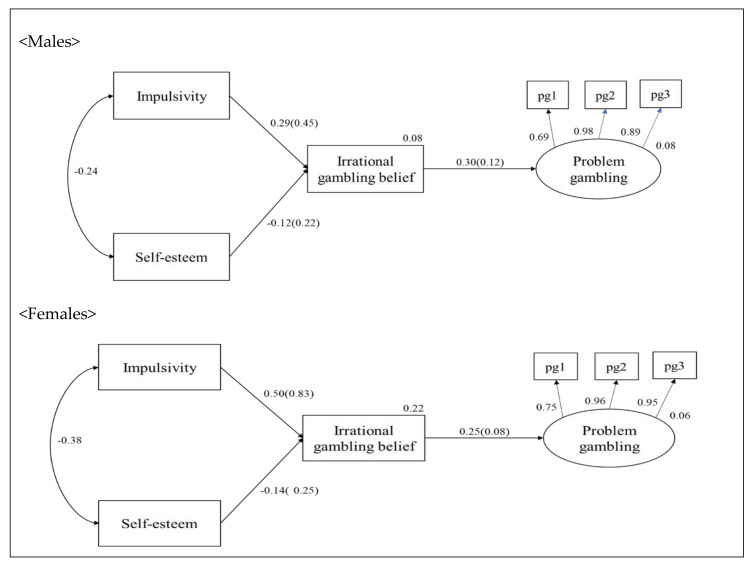
Standardized coefficients (unstandardized coefficients) for a model of the relationship between impulsivity and self-esteem, irrational gambling belief, and problem gambling—estimates for males and females. Note: All coefficients are significant at *p* < 0.05.

To evaluate the moderating effect of gender differences, a multi-group test was carried out. Two groups were created, one for male college students and one for female college students, and then a multi-group test was executed through AMOS. To determine which model best accounted for the data of the gender groups, a series of nested models were estimated. The fit indices of the models are shown in Table 7. In Step 1, all parameters were freely estimated for each group. In Step 2, the invariance of the factor loadings was examined for equality across groups, and there was a significant change in the fit indices of the model. In order to conduct partial metric invariance verification, the *χ*^2^ value and the difference in degrees of freedom and goodness of fit were compared by releasing one factor coefficient constraint of the two groups. The equality constraint of the pg3 indicator was released. The metric invariance was partially satisfied. In Step 3, structural path invariance was examined by constraining the relationships between the latent variables to equality across groups. As a result, there was a significant change in the fit indices of the model (Δ*χ*^2^ = 19.72, Δ*df* = 1, *p* < 0.05), suggesting that the two groups differ significantly in the structural relationships between their latent variables (See Table 7). In addition, unstandardized path coefficients were used for comparing each group in the multiple group analysis. Standardized path coefficients can be used to determine the relative importance of the coefficient. However, it is impossible to compare the coefficients of each sample when the samples are separated. Table 8 shows that gender moderates the relationship between impulsivity, self-esteem, irrational gambling belief, and problem gambling. These findings indicate that problem gambling is related to impulsivity, self-esteem, and irrational gambling belief in different ways for males and females. H7 (Gender moderates the links between impulsivity, self-esteem, irrational gambling belief, and problem gambling) was thus accepted (see Figure 3). For both males and females, impulsivity and self-esteem were related to irrational gambling belief, and irrational gambling belief was positively related to problem gambling. For females, such that irrational gambling belief functioned as a mediator variable between impulsivity and self-esteem and problem gambling (β = 0.13, *p* < 0.001; β = 0.04, *p* < 0.05). For males, in contrast, impulsivity was positively related to irrational gambling belief, and irrational gambling belief was positively related to problem gambling (β = 0.09, *p* < 0.01); the mediating effect of irrational gambling belief between self-esteem and problem gambling was not significant (β = 0.04, *p* > 0.05). The present findings thus indicate that gender moderates the relationships between impulsivity, self-esteem, and irrational gambling belief and problem gambling.

## 4. Discussion

This study aimed to explore whether the relationships between impulsivity, self-esteem, irrational gambling belief, and problem gambling are different for males and females.

First, impulsivity had a significant positive relationship with irrational gambling belief, and irrational gambling belief had a significant positive relationship with problem gambling. These results underscore the importance of impulsivity and irrational gambling belief in problem gambling [10]. Previous studies showed that adults with the characteristics of impulsiveness had more symptoms of problem gambling than those who were less impulsive [10,11,12,48]. Problem gambling is a mental disorder and is associated with dysfunction in the cognitive domains that control impulsive behavior [7]. Impulsive people tend to act without fully realizing the consequences of their actions. Therefore, psychosocial intervention should focus on impulsiveness as a risk factor or vulnerable personality trait. This research also supports the long-standing hypothesis that irrational beliefs are a continuous and powerful cognitive property of pathological gambling [49]. Therefore, the treatment of problem gamblers should also focus on modifying irrational thinking and behavior of the program. However, self-esteem was not shown to have a significant relationship with problem gambling in this research. The present results are inconsistent with previous studies on the relationships between self-esteem and problem gambling [15,17]. Further studies should explore the relationship between self-esteem and problem gambling more comprehensively, including the relevant mediating and moderating variables.

Second, comparing the fitness of the research model and the alternative model, the alternative model (full mediation model) was found to be more appropriate; the direct path from impulsivity to problem gambling and the direct path from self-esteem to problem gambling were removed. Impulsivity has a direct effect on irrational gambling beliefs, and irrational gambling beliefs have a direct effect on problem gambling. Moreover, impulsivity has an indirect effect on problem gambling via irrational gambling beliefs. The results indicate that an individual’s impulsiveness affects his or her problem gambling through irrational beliefs rather than directly affecting problem gambling. In other words, the more impulsive college students are, the more likely they are to have irrational gambling beliefs, and this higher level of irrational gambling belief tends to cause problem gambling. These findings suggest the importance of cognitive characteristics in the impact of individual emotional characteristics on gambling problems [29]. It is widely known that problem gamblers have irrational and distorted beliefs about gambling [23,26,29]. It is not easy to change the inclinations or behaviors of problem gamblers all at once. In comparison, prior studies have reported that problem gamblers have changed their distorted thinking on gambling through education or training [50]. Therefore, it is necessary to provide systematic psychosocial programs to change problem gamblers’ distorted beliefs about games and, above all, to provide problem gamblers psychological treatment based on a cognitive–behavioral approach to provide opportunities to apply this knowledge to gambling settings [51].

Third, the relationships between impulsivity, self-esteem, irrational gambling belief, and problem gambling differed for males and females. Our findings thus indicate that gender moderates the relationships between impulsivity, self-esteem, and irrational gambling belief, as well as problem gambling. For females, both impulsivity and self-esteem were related to irrational gambling belief, and irrational gambling belief was positively related to problem gambling such that irrational gambling belief functioned as a mediator variable between impulsivity, self-esteem, problem gambling. For males, in contrast, impulsivity was positively related to irrational gambling belief, and irrational gambling belief was positively related to problem gambling. These results were partially consistent with previous studies, which showed that male gamblers have more irrational beliefs than female gamblers and are more likely than females to experience gambling problems [52]. Therefore, different strategies for male and female problem gamblers may be necessary to maximize the efficacy of interventions [53]. Interventions for female problem gamblers should be focused on lowering impulsiveness and increasing self-esteem to lower female’s irrational gambling beliefs, while interventions for male problem gamblers should focus on lowering impulsiveness to reduce irrational gambling beliefs. In particular, the findings suggest that efforts are needed to reduce irrational gambling beliefs by encouraging and support self-esteem to prevent female gambling problems. Considering the fact that self-esteem was a protective factor influencing the addiction of gambling industry users [54], efforts should be made to strengthen the self-esteem of female problem gamblers by identifying the cause of their low self-esteem. However, it was difficult to pinpoint the cause of gender differences in the relationships between impulsiveness, self-esteem, and irrational beliefs and problem gambling in this study. In fact, the consequences of gambling behavior could be the cause of low self-esteem and vice versa. Further research is needed to provide more practical information to prevent and address problem gambling by verifying models, including the environmental and personal variables that affect problem gambling.

## 5. Conclusions

Due to increased access to gambling platforms and increasing gambling opportunities in Korea, the prevention of problem gambling is utmost; it is necessary to verify what influences male and female college students’ problem gambling. Considering that the emotional characteristics (such as impulsiveness and self-esteem) of college students influenced problem gambling via irrational beliefs, efforts to transform college students’ unreasonable beliefs into rational ones are paramount. This study also suggested that gender-specific intervention efforts are needed. For females, greater impulsivity and lower self-esteem predicted higher irrational gambling belief, and higher irrational gambling belief predicted greater problem gambling. For males, greater impulsivity predicted higher irrational gambling belief, and higher irrational gambling belief predicted greater problem gambling. The intervention of problem gambling for males should focus on controlling impulsivity and changing irrational beliefs, and psychosocial intervention of problem gambling for females should focus on strengthening psychological characteristics such as self-esteem.

## Figures and Tables

**Table 1:** Descriptive statistics of the main variables (*n* male = 259, *n* female = 304).

Variables	Theoretical Range	Range	M (SD)			Skewness	Kurtosis
			Male	Female	Total		
Impulsivity	1–4	1–4	1.97 (0.58)	2.07 (0.54)	2.02 (0.59)	−0.07	−0.41
Self-esteem	1–4	1.30–4	3.00 (0.50)	2.86 (0.52)	2.92 (0.52)	0.41	−0.51
Irrational gambling belief	1–5	1–4.7	2.20 (0.90)	1.85 (0.82)	2.01 (0.88)	0.38	−0.97

**Table 2:** A comparison of PGSI on by gender (*n* male = 259, *n* female = 304).

	Subgroup	M (SD)			*χ* ^2^	*p*
		Male	Female	Total		
PGSI *	Non-problem	153 (59.1)	235 (77.3)	388 (68.9)	23.92	<0.001
	Low risk	51 (19.7)	41 (13.5)	92 (16.3)		
	Moderate risk	36 (13.9)	17 (5.6)	53 (9.4)		
	Problem gambler	19 (7.3)	11 (3.6)	30 (5.3)		

* PGSI (Problem gambling severity total score).

**Table 3:** Correlations among the main variables.

Variables	Impulsivity	Self-Esteem	Irrational Gambling Belief
Self-esteem	−0.32 ***	-	-
Irrational gambling belief	0.25 ***	−0.02	-
Problem gambling	0.15 **	−0.02	0.33 ***

** *p* < 0.01. *** *p* < 0.001.

**Table 4:** Model fitness index for the hypothesized model and the alternative model.

Model	*χ* ^2^	*df*	*p*	NFI	CFI	RMSEA	Δ*χ*^2^
Hypothesized model	46.44	6	0.00	0.97	0.97	0.80	-
Alternative model (Full mediation model)	51.04	8	0.00	0.97	0.97	0.70	4.60

*df*: degree of freedom; NFI: Normed fit index; CFI: Comparative Fit Index; RMSEA: Root Mean Square Error of Approximation.

**Table 5:** Regression weights of the final model.

Description	Estimate (Unstandardized)	Estimate (Standardized)	S. E	C. R
Impulsivity → Irrational gambling belief	0.44	0.29 ***	0.06	6.73
Self-esteem → Irrational gambling belief	0.20	−0.12 *	0.07	2.71
Irrational gambling belief → Problem gambling	0.10	0.28 ***	0.02	6.37

* *p* < 0.05. *** *p* < 0.001. S. E.: Standard Error; C. R.: Critical Ratio.

**Table 6:** Standardized direct and indirect effect.

	Direct Effect	Indirect Effect	Total Effect
Impulsivity → Irrational gambling belief	0.29 ***		0.29 ***
Self-esteem → Irrational gambling belief	0.12		0.12
Impulsivity → problem gambling		0.08 **	0.08
Self-esteem → problem gambling		0.03 *	0.03
Irrational gambling belief → Problem gambling	0.28 ***		0.28 ***

* *p* < 0.05, ** *p* < 0.01, *** *p* < 0.001.

**Table 7:** Fit indices for the nested sequence of models in multigroup analysis.

Model	*χ* ^2^	*df*	*p*	RMSEA	NFI	CFI	Δ*χ*^2^	Δ*df*
Configural invariance	50.37	16	0.00	0.06	0.98	0.98		
Metric Invariance	60.98	18	0.00	0.07	0.97	0.97	10.61	2
Partial Metric Invariance	51.47	17	0.06	0.06	0.98	0.98	0.10	1
Scalar invariance	71.19	18	0.00	0.07	0.96	0.96	19.72	1

*df*: degree of freedom; NFI: Normed fit index; CFI: Comparative Fit Index; RMSEA: Root Mean Square Error of Approximation.

**Table 8:** Unstandardized estimates and standard errors of structural parameters in the two groups.

Description	Male			Female		
	Estimates	S. E.	C. R.	Estimates	S. E.	C. R.
Impulsivity → Irrational gambling belief	0.44 ***	0.09	4.73	0.83 ***	0.04	24.20
Self-esteem → Irrational gambling belief	0.22 *	0.10	2.01	0.25 **	0.09	2.77
Irrational gambling belief → Problem gambling	0.13 ***	0.03	4.91	0.08 ***	0.02	4.32

* *p* < 0.05, ** *p* < 0.01. *** *p* < 0.001. S. E.: Standard Error; C. R.: Critical Ratio.

## Data Availability

Not applicable.
